# Thoracic Segmental Spinal Anesthesia (TSSA) Recovery Score: A Proposed Tool for Assessing Discharge Readiness After Thoracic Segmental Spinal Anesthesia

**DOI:** 10.7759/cureus.106006

**Published:** 2026-03-27

**Authors:** Imran Ahmed Khan, Naresh Paliwal, Geetanjali Singhal

**Affiliations:** 1 Anesthesiology and Public Health, Keshav Memorial Charity (KMC) Medical College and Hospital, Maharajganj, IND; 2 Anesthesiology, Dr. Panjabrao Deshmukh Memorial Medical College, Amravati, IND; 3 Anesthesiology, Jaipur National University, Jaipur, IND

**Keywords:** ambulatory surgical procedures, checklist, hemodynamics, patient discharge, patient safety, spinal puncture

## Abstract

Thoracic segmental spinal anesthesia (TSSA) has emerged as a promising regional anesthesia technique for diverse surgical procedures, offering targeted blockade, stable hemodynamics, and rapid postoperative recovery. Despite increasing adoption, standardized discharge criteria specific to TSSA remain absent, leading to variability in clinical practice and potential safety concerns. To address this gap, we propose a simple four-parameter scoring system designed to evaluate hemodynamic stability, upper and lower sensory block regression, and motor recovery. Each parameter is graded from 0 to 2, yielding a maximum score of 8. A score ≥7, combined with standard recovery criteria, indicates readiness for discharge. This scoring system provides a bedside structured approach for assessing recovery following TSSA. Prospective validation studies are warranted to confirm its reliability, generalizability, and clinical utility across diverse patient populations.

## Introduction

Thoracic segmental spinal anesthesia (TSSA) has emerged as a valuable neuraxial anesthesia technique for selected surgical procedures [1]. Unlike conventional lumbar spinal anesthesia (LSA), TSSA produces a more localized blockade, called a segmental blockade, depending on the level of spinal puncture, drug doses, and other factors. This segmental approach allows anesthesiologists to achieve adequate surgical anesthesia while minimizing the extent of neuraxial blockade [2]. TSSA is most commonly performed with low-dose hyperbaric or isobaric local anesthetics (LA), with or without an adjuvant. Hyperbaric preparations produce a faster onset of sensory and dense motor blocks. However, hyperbaric formulations need careful patient positioning to guide the block extent. Patient position and gravity do not affect isobaric solutions [3].

Several potential advantages of TSSA have been described, including improved hemodynamic stability, reduced anesthetic drug requirements, enhanced analgesia, and potentially faster postoperative recovery [4]. With increasing clinical experience and expanding indications for TSSA, patients receiving it may recover rapidly and become suitable for early postoperative discharge [5].

TSSA differs from LSA in the level and distribution of neuraxial blockade, which may influence the timeline and characteristics of postoperative recovery. Despite the growing interest in TSSA, there remains no standardized scoring system specifically designed to assess discharge readiness following TSSA. However, a SAFE-TSSA safety checklist has been recently published [6]. Postanesthesia recovery is typically assessed using standardized tools such as the Modified Aldrete Score [7]. While this widely adopted scoring system assesses recovery following anesthesia, it does not fully incorporate recovery specific to neuraxial anesthesia.

Standardized recovery assessment tools are important for several reasons. They provide objective criteria for discharge decisions, reduce variability in clinical practice, improve patient safety, and facilitate research into postoperative recovery profiles [8]. Therefore, we propose the TSSA Recovery Score (TRS) as a simple, practical bedside tool to assist anesthesiologists in evaluating patient readiness for discharge after procedures performed under TSSA.

## Technical report

Development of the TSSA Recovery Score

The proposed scoring system was designed to be simple, clinically practical, and easily applicable in the operating room or postanesthesia care unit (PACU). This scale evaluates hemodynamic parameters and three TSSA-specific domains: regression of upper sensory block, regression of lower sensory block, and recovery of lower limb motor function (Table 1). Because TSSA produces a segmental thoracic block, recovery assessment should consider regression of both the cephalad and caudal sensory levels. The upper sensory level is clinically relevant because excessive cephalad spread may affect thoracic dermatomes involved in respiratory mechanics and sympathetic blockade. On the other hand, regression of the lower sensory level reflects the integrity of lumbar and sacral neural function, which is important for safe ambulation and neurological recovery. Sensory regression was assessed bilaterally using loss of cold sensation, and motor recovery was evaluated using the Bromage scale. Each parameter was assigned a score ranging from 0 to 2, allowing rapid bedside assessment while maintaining simplicity. The four parameters were selected through a three-step process: 1) review of the TSSA literature focusing on recovery profiles, 2) clinical experience of the authors, and 3) alignment with the domains highlighted in the SAFE-TSSA expert consensus checklist and bundle for TSSA [9]. Hemodynamic stability was included because cardiovascular instability is the most common perioperative concern. Each item was graded 0-2 to maintain simplicity and allow rapid bedside use.

**Table 1:** TSSA Recovery Score parameters Parameters and scoring criteria were derived from literature on TSSA recovery, and author consensus aligned with the SAFE-TSSA checklist BP, blood pressure; HR, heart rate; T, thoracic; L, lumbar; TSSA, thoracic segmental spinal anesthesia

Parameter	Score 2	Score 1	Score 0
Hemodynamic stability	BP and HR within 20% of baseline	BP or HR 20%-30% deviation from baseline	BP or HR >30% deviation or requiring vasopressors
Upper sensory regression	Upper level ≤T6	T4-T5	≥T3
Lower sensory regression	Lower level ≥T12	L1	≤L2
Motor recovery	Bromage 0 (full movement)	Bromage 1-2	Bromage 3

Table 2 provides an interpretation of TRS, where the minimum value could be 0 and the maximum score is 8. A total score of ≥7 was considered adequate recovery and discharge readiness. A score of 7-8 ensures that no parameter is scored 0 (i.e., no major deviation) and at most one parameter is scored 1. This conservative cutoff balances patient safety with the rapid-recovery profile of TSSA and is conceptually similar to the Modified Aldrete Score, in which a score <9 typically mandates continued observation.

**Table 2:** Interpretation of the TSSA recovery score TSSA, thoracic segmental spinal anesthesia

Total score	Interpretation	Clinical action
7-8	Adequate recovery; patient ready for discharge	Proceed to standard discharge checklist
5-6	Near recovery	Continued observation and reassessment
≤4	Incomplete recovery	Further monitoring required

The proposed discharge threshold and dermatomal cutoffs require validation in prospective studies; a pilot validation study with an adequate sample size and statistical testing is planned prior to any recommendation for routine clinical use.

## Discussion

Although TSSA offers greater hemodynamic stability than LSA or general anesthesia (GA) in selected patients, transient hypotension and bradycardia still occur, and high or total spinal block remains a rare but serious risk [10,11]. A recent meta-analysis found higher odds of hypotension and bradycardia with TSSA compared to GA [12]. These complications, combined with the absence of any TSSA-specific discharge criteria, create a clear knowledge gap that may delay safe early discharge or expose patients to unnecessary risk. The recently published SAFE-TSSA checklist addresses intraoperative safety but does not cover postoperative recovery assessment.

The proposed TRS offers several advantages. It is simple and easy to apply, requiring only basic clinical assessment without specialized equipment. The scoring system can be completed within minutes in the PACU. The score is also specific, incorporating parameters that reflect recovery from TSSA.

The TRS may have several practical applications in clinical practice. First, the scoring system provides a standardized approach for evaluating postoperative recovery following TSSA. A structured scoring system may help reduce variability in practice and improve consistency among clinicians.

Second, the score may be particularly useful in ambulatory surgery, where early discharge is a desired goal. By providing clear discharge thresholds, the scoring system may support enhanced recovery protocols and improve patient flow. Third, the score may serve as a research tool for future studies examining recovery profiles after TSSA. Finally, the scoring system emphasizes neurological recovery parameters such as sensory block regression and motor function, which are particularly relevant in neuraxial anesthesia but are not fully addressed in traditional recovery scoring systems.

**Figure 1:**
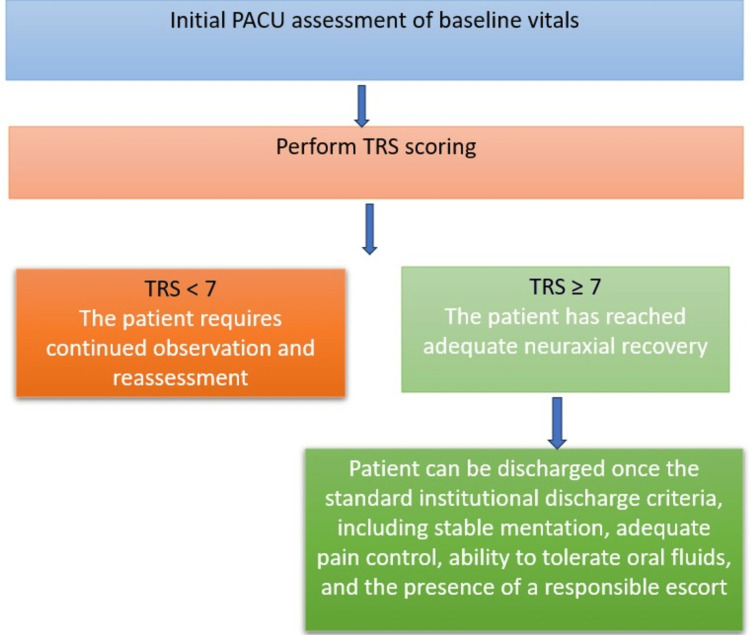
Discharge workflow after TSSA PACU, postanesthesia care unit; TSSA, thoracic segmental spinal anesthesia; TRS, TSSA Recovery Score

Although the scoring system provides an objective framework for assessing TSSA recovery, it is not meant to replace traditional recovery criteria. Other safety criteria should also be verified before discharge, including an awake and oriented patient, stable hemodynamics, tolerating oral fluids, no urinary retention or bladder discomfort, and a responsible adult escort available [13]. Hyperbaric LA may prolong sacral block; therefore, urinary retention must be assessed separately (bladder scan or time to void) before discharge [14]. TRS is intended to complement, rather than replace, traditional readiness criteria, such as the Modified Aldrete Score. Figure 1 provides a clinical workflow for TSSA recovery assessment.

The present report introduces a conceptual scoring system and therefore has several limitations. The TRS has not yet been validated in clinical practice. Prospective studies are required to determine its reliability, sensitivity, and specificity in predicting safe discharge. Standardized training for PACU staff, specifically regarding the assessment of sensory levels using loss of cold sensation and motor function via the Bromage scale, is essential for the consistent application of the TRS and reduced interobserver variability. Finally, the proposed discharge threshold requires validation in diverse patient populations. The score may perform differently in elderly patients and those with comorbidities or high-risk surgeries.

Future research should focus on validating the TRS in different clinical settings. Comparative studies may also evaluate the performance of the proposed score against established recovery tools such as the Modified Aldrete Score. Such investigations will help determine whether the scoring system can become a standardized recovery assessment tool for patients undergoing surgery under TSSA.

## Conclusions

The TRS is a proposed bedside scoring system designed to assess recovery following TSSA. By incorporating hemodynamic stability and neuraxial recovery parameters, including regression of sensory block and restoration of motor function, TRS provides a structured framework for assessing discharge readiness. Although further validation is necessary, this scoring system may contribute to standardized postoperative assessment and facilitate safe discharge practices in patients receiving TSSA.
